# Comparative analysis of gene expression patterns in the arthropod labrum and the onychophoran frontal appendages, and its implications for the arthropod head problem

**DOI:** 10.1186/s13227-016-0064-4

**Published:** 2017-01-03

**Authors:** Ralf Janssen

**Affiliations:** Department of Earth Sciences, Palaeobiology, Uppsala University, Villavägen 16, 75236 Uppsala, Sweden

**Keywords:** Arthropod head problem, Onychophora, Arthropoda, Labrum, Evolution

## Abstract

The arthropod head problem has troubled scientists for more than a century. The segmental composition of the arthropod head, homology of its appendages, and especially the nature of the most anterior region of the head are still, at least partially, unclear. One morphological feature of the head that is in the center of current debate is the labrum (upper lip), a fleshy appendicular structure that covers the arthropod mouth. One hypothesis is that the labrum represents a fused pair of protocerebral limbs that likely are homologous with the frontal appendages (primary antennae) of extant onychophorans and the so-called great appendages of stem arthropods. Recently, this hypothesis obtained additional support through genetic data, showing that *six3*, an anterior-specific gene, is exclusively expressed in the arthropod labrum and the onychophoran frontal appendages, providing an additional line of evidence for homology. Here I present data that put this finding into perspective. The outcome of my study shows that the homologization of a morphological structure by the expression of a single genetic factor is potentially misleading.

**Electronic supplementary material:**

The online version of this article (doi:10.1186/s13227-016-0064-4) contains supplementary material, which is available to authorized users.

## Background

“The endless dispute” [1] describes one of the most long-standing zoological problems: the segmental composition and homology of the anterior of the arthropod head and its appendages. Since Rempel’s assumptions that these problems would likely never be solved, quite some progress has been made, and homology of head segments has been solved by the investigation of innervation patterns and Hox gene expression patterns (reviewed in [2–4]). A remaining problem, however, is the unclear nature of the pre-antennal (pre-cheliceral in Chelicerata) region, and the nature of the labrum, the upper lip of arthropods. The labrum represents an enigmatic structure of unclear origin and homology. It is discussed controversially if the labrum is an appendicular structure, and if it is serially homologous to the other limbs. Even more unclear is its segmental affinity (if any) (reviewed in, e.g., [1, 3, 5]).

Most authors agree that the labrum represents a pair of fused limbs, based on the fact that it originates as two independent buds that fuse later during ontogeny, and partially conserved gene expression patterns (e.g., [6–15]). Nevertheless, some authors doubt its homology with the other limbs and instead suggest that the labrum may have evolved independently, and that the apparent genetic similarities are the result of convergent evolution rather than homology [3, 11–13, 16, 17].

Onychophorans represent the likely sister group to arthropods or at least form a closely related outgroup (depending on the still unclear phylogenetic position of Tardigrada) (e.g., [18–21]). Morphological studies and gene expression analysis of conserved Hox gene patterning in extant arthropods and onychophorans revealed that the onychophoran frontal appendages are not homologous with the mandibulate antennae (despite their morphological and functional similarity) but instead represent more anterior appendages [22–26]. Therefore, the idea has been put forward that the labrum is homologous with the frontal appendages of extant onychophorans and stem-group lobopodians and the great appendages of stem-group arthropods (e.g., [27–30]), but see [3, 16, 17] for another homology hypothesis.

In order to support homology of the frontal appendages with the labrum, Scholtz and Edgecombe [3] suggested to search for genes that are exclusively expressed in both structures, but not in any of the other appendages.

Recently, one such factor, *six3*, was revealed. *six3* controls patterning of the so-called anterior median region (AMR) in bilaterian animals ([31–37]), and many of the genes that are expressed in the AMR are also expressed in the arthropod labrum offering the opportunity to find more shared specific factors that could support homology of the labrum with the frontal appendages.

In order to put the homology test (as suggested by [3]) to the test, in the current paper, expression patterns of AMR/labrum-patterning genes have been investigated in the onychophoran *Euperipatoides kanangrensis* and, in order to obtain a more solid basis for comparison, also in the myriapod *Glomeris marginata*. The data indicate that the AMR is principally conserved in onychophorans, but the ambiguous nature of the results with respect to expression in the frontal appendages indicates that homologization of the labrum with the frontal appendages based on the expression of a single gene, such as *six3*, is problematic.

## Methods

### Embryo collection, fixation and developmental staging

Onychophoran embryos were dissected from pregnant females, prepared for in situ hybridization experiments, and staged as described in [38, 39]. Myriapod embryos were collected, fixed and staged as described in [40].

### Gene cloning

Total RNA was extracted (TRIzol, Invitrogen, Carlsbad, CA) and reverse transcribed into cDNA (SuperScriptII first-strand synthesis system for RT-PCR, Invitrogen). Gene fragments were isolated by means of RT-PCR with gene-specific primers (Additional file 1: Table S1) based on sequenced embryonic transcriptomes. All gene fragments were cloned into pCRII vectors (TA cloning kit dual promoter, Invitrogen) and sequenced by a commercial sequencing service (Macrogen, Seoul, South Korea). Newly recovered sequences are available under accession nos. LT560250 (*Ek*-*hbn*), LT560249 (*Ek*-*FoxQ2*), LT560252 (*Ek*-*nkx2.1/scro*), LT560251 (*Ek*-*rx*), LT560253 (*Ek*-*vsx/chx*), LT560255 (*Gm*-*hbn*), LT560254 (*Gm*-*FoxQ2*), LT560256 (*Gm*-*nkx2.1/scro*).

### In situ hybridization

Whole-mount in situ hybridization of *E. kanangrensis* and *G. marginata* embryos was performed as in [41]. Digoxigenin-labeled RNA probes were transcribed from the cloned gene fragments. Cell nuclei were stained by incubation in 2 µg/ml of the fluorescent dye 4-6-diamidino-2-phenylindole (DAPI) in phosphate-buffered saline with 0.1% Tween 20 (PBST) for 20–30 min at room temperature (Additional file 2: Table S2).

### Data documentation

Photographs were taken with a Leica DC100 digital camera under a Leica dissection microscope. The image processing software Adobe PHOTOSHOP CS2 (v. 9.0.1 for Apple Macintosh) was used for linear corrections of brightness, contrast and color values.

### Phylogenetic analysis

Protein sequences of the complete open reading frames of the homeodomain-encoding genes Rx, Vsx/Chx, Hbn and Nkx2.1/Scro of the fly *Drosophila melanogaster*, the beetle *Tribolium castaneum*, *Euperipatoides* and *Glomeris*, have been aligned using T-Coffee [42]. As closely related outgroup sequences serve ventral nervous system defected (Vnd) from *Drosophila* and *Tribolium* as well as aristaless (Al) from *Drosophila*. The same has been done for the forkhead domain of all known *Drosophila* and *Strigamia maritima* Fox genes [43, 44] and FoxQ2 from *Glomeris* and *Euperipatoides*. The Fox2 gene of *Saccharomyces cerevisiae* serves as outgroup sequence. In both cases, Bayesian phylogenetic analyses were performed with MrBayes [45] using a fixed WAG amino acid substitution model with gamma-distributed rate variation across sites (with four rate categories). An unconstrained exponential prior probability distribution on branch lengths and an exponential prior for the gamma shape parameter for among-site rate variation were applied. The final topology was estimated using 1,000,000 cycles for the MCMCMC (metropolis-coupled Markov chain Monte Carlo) analysis with four chains and the chain heating temperature set to 0.2. The Markov chain was sampled every 200 cycles. Clade support was assessed with posterior probabilities computed with MrBayes.

## Results

### Phylogenetic analysis

**Fig. 1:**
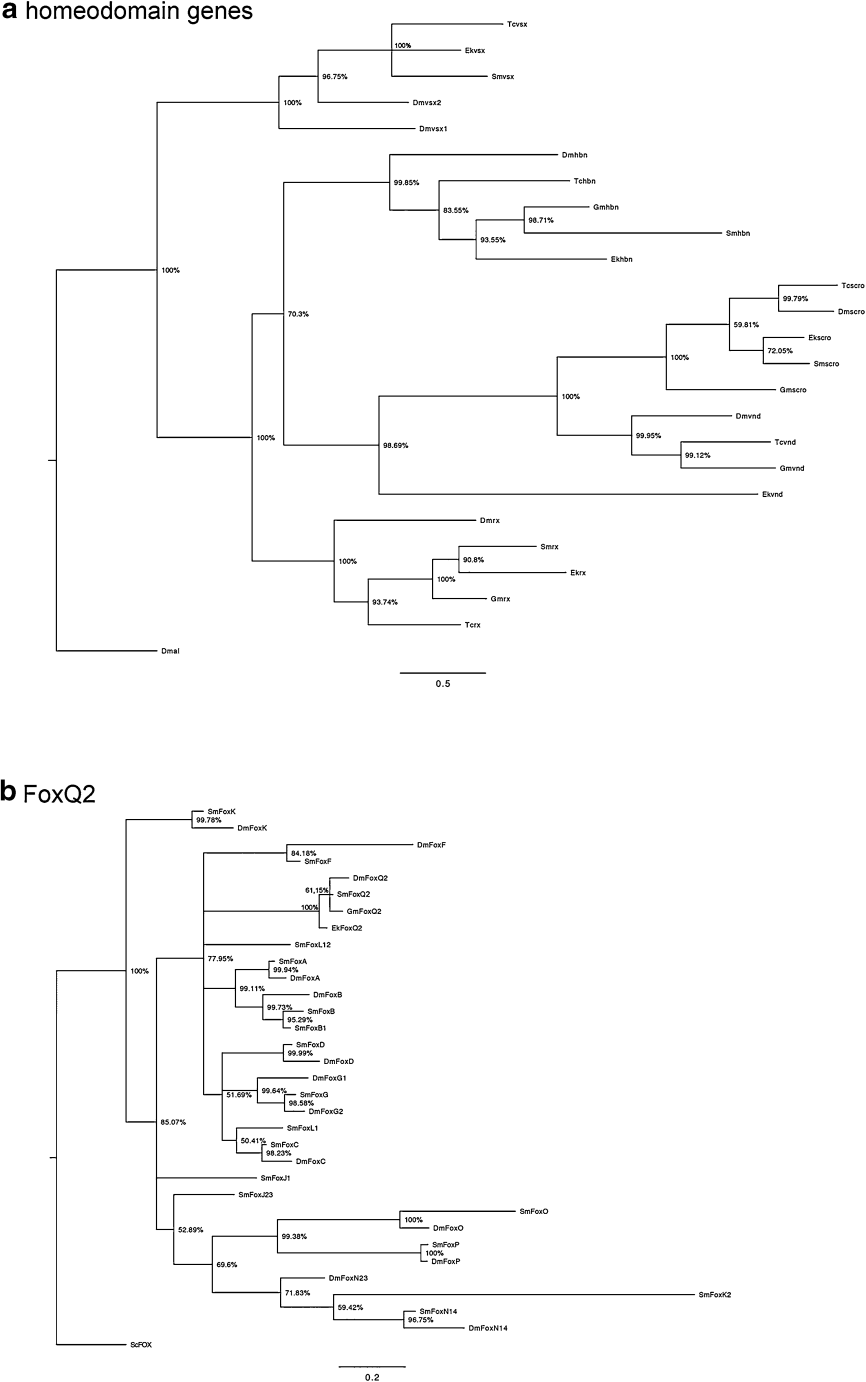
Phylogenetic analysis of homeodomain genes and FoxQ2. Species abbreviations: Ek, *Euperipatoides kanangrensis* (Onychophora); Dm, *Drosophila melanogaster* (Hexapoda: Diptera); Gm, *Glomeris marginata* (Myriapoda: Diplopoda) Tc, *Tribolium castaneum* (Hexapoda: Coleoptera); Sm, *Strigamia maritima* (Myriapoda: Chilopoda). Gene abbreviations: al, aristaless; Fox, forkhead box gene; rx, retinal homeobox; scro, scarecrow; vnd, ventral nervous system defective; vsx, visual system homeobox. See text for further information

 Predicted onychophoran orthologs of visual system homeobox (Vsx/Chx), retinal homeobox (Rx), Scarecrow (Scro), homeobrain (Hbn) and FoxQ2 cluster with high reliability with their arthropod orthologs (Fig. 1a, b). The putative ortholog of ventral nervous system defective (Vnd) branches at the base of arthropod Vnd and arthropod + onychophoran Scro. Expression analysis revealed that *Euperipatoides vnd* is not expressed in the head, but is, like arthropod vnd genes, predominantly expressed in the ventral nerve cord (not shown).

### Expression patterns

**Fig. 2:**
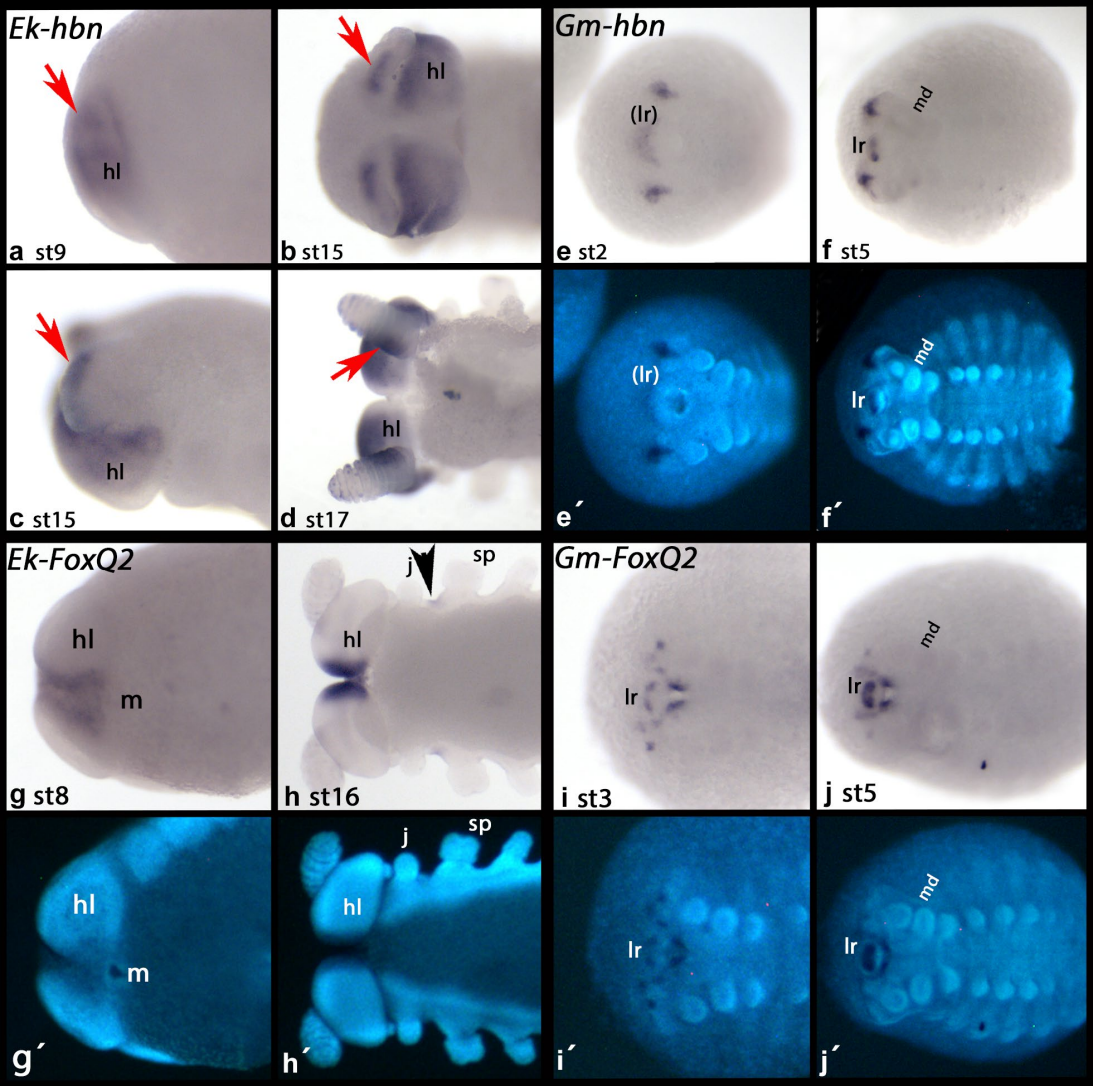
Expression of *hbn* and *FoxQ2.* In *all panels* anterior is to the left. Ventral view, if not indicated otherwise. **a–d** *Euperipatoides hbn*. **a**, **c** Lateral view. **d** Dorsal view. *Arrows* point to expression in the frontal appendages. **e**, **f** *Glomeris hbn*. **g**, **h** *Euperipatoides FoxQ2*. *Arrowhead marks* expression between the jaw and the slime papilla. **i**, **j** *Glomeris FoxQ2*. *lr* primordium of the labrum, *hl* head lobe, *j* jaw, *lr* labrum, *m* mouth, *md* mandible, *sp* lime papilla

*Euperipatoides homeobrain* (*Ek*-*hbn*) is exclusively expressed in the head, including the developing frontal appendages. The latter is restricted to ventral and proximal tissue (Fig. 2), very similar to the expression of *six3* [5]. *Glomeris hbn* is expressed in form of two patches in the very anterior of the head and in a domain in the primordium of the labrum (Fig. 2). At later stages, this latter expression is located dorsally in the developing labrum and thus in a comparable pattern as seen in the onychophoran (if the rotation theory put forward by [10] holds true).

*Euperipatoides FoxQ2* (*Ek*-*FoxQ2*) is expressed in the ventral region of the head lobes, anterior to the mouth, but not the frontal appendages. At later stages, it is expressed also in a patch-like domain at the base between the jaws and the slime papillae (Fig. 2). *Glomeris FoxQ2* is expressed in a complex pattern in the anterior of the head including the labrum.

**Fig. 3:**
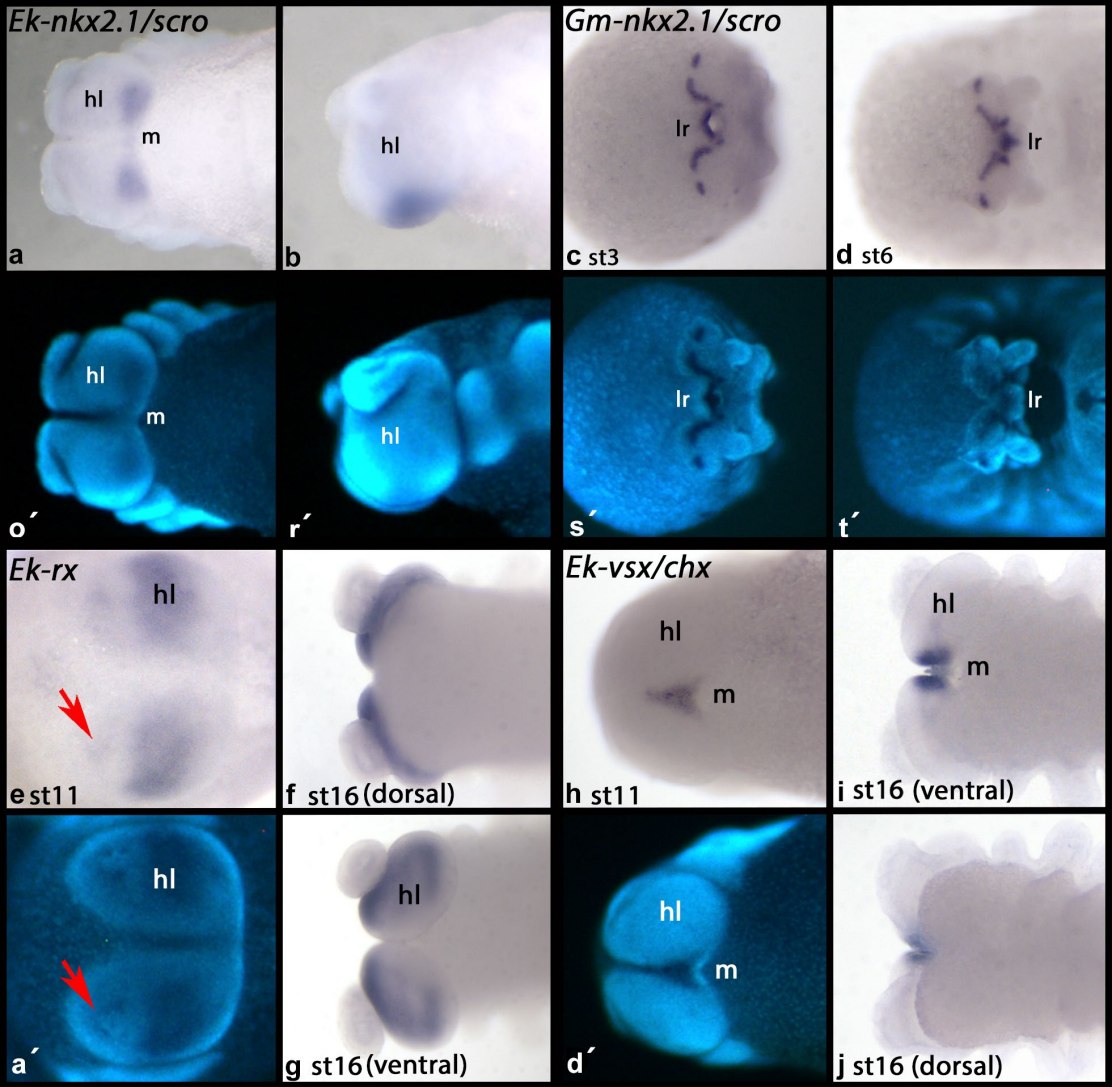
Expression of *nkx2.1/scro*, *rx* and *vsx/chx.* In all panels anterior is to the *left*. Ventral view, if not indicated otherwise. **a**, **b** *Euperipatoides nkx2.1/scro*. **b** Lateral view. **c**, **d** *Glomeris nkx2.1/scro*. **c** Anterior view. **e–g** Expression of *Euperipatoides rx.* **a** anterior view. **h–j** Expression of *Euperipatoides vsx/chx*. **j** Dorsal view. Abbreviations as in Fig. 2

*Euperipatoides scarecrow* (*Ek*-*nkx2.1/scro*) is expressed in ventral and posterior tissue of the head lobes, but not in the frontal appendages (Fig. 3). In contrast to that, *Glomeris nkx2.1/scro* is expressed in a complex pattern in the anterior head as well as in the labrum (Fig. 3).

*Euperipatoides retinal homeobox* (*Ek*-*rx*) is exclusively expressed in the head lobes. At early developmental stages, it is expressed weakly and transiently in the frontal appendages. Later, however, it is not expressed in the frontal appendages anymore (Fig. 3).

*Euperipatoides visual system homeobox* (*Ek*-*vsx/chx*) is expressed in the very ventral region of the head lobes, anterior of the mouth. This domain resembles very much the domain in which *FoxQ2* is expressed. However, expression of *vsx/chx* is slimmer and does not reach as far toward anterior as that of *FoxQ2*. Also, *vsx/chx* is not expressed posterior to the head lobes (Fig. 3).

## Discussion

### Putting the homology test to the test: taking a look beyond six3

 In their very much noticed review article Scholtz and Edgecombe [3] suggested to search for genes that are specifically expressed in the labrum and the frontal appendages (but not in any other appendage type) since those patterns would likely serve as direct evidence for homology: “…, if peculiarities of gene expression in the labrum, which are absent in trunk limbs of euarthropods, find their correspondence in that of onychophoran ‘antennae,’ we could have direct evidence for homology between these two structures” [3].

Possibly as a direct consequence, when the *test* was undertaken, as expression of *six3* was found in both, the arthropod labrum and the onychophoran frontal appendages, some authors took this as a possible, or even convincing, homology criterion [5, 23, 46, 47].

However, the past has shown that it is problematic to use a single gene as strong evidence for homology (discussed in [48]), as exemplified by the use of, e.g., the segment polarity gene *engrailed* (*en*) alone and out of context for the identification of head segments (discussed in, e.g., [4]), or the use of *distal*-*less* (*Dll*) alone, and out of its genetic context, for the identification of serially homologous appendages (discussed in, e.g., [9]). *en* and *Dll* are embedded in a conserved gene regulatory network as segmentation and appendage-patterning genes, respectively, but they are also expressed in other, non-homologous structures such as anal valves and sensory bristles. The same problem may occur with the use of *six3* as a marker for the labrum/frontal appendages. In fact, the role of *six3* in appendage patterning is not exclusive for the frontal appendages of onychophorans and the arthropod labrum, but it is also expressed in other sensory appendages in at least myriapods [33, 36], although this may be explained best by convergent evolution rather than an ancestral role of *six3* in patterning these limbs.

However, as Aristotle (384-322 BC) pointed out “One swallow does not a summer make, …”, likewise the expression of one gene, not even that of the famous *six3*, *Dll*, or *en* necessarily defines the evolutionary nature of a morphological structure.

The data obtained in this study support this concern as they are ambiguous (Figs. 2, 3). While some genes appear to support homology of the arthropod labrum with the onychophoran frontal appendages (i.e., *six3* and *hbn*) (Fig. 2) [5, 37], others do not, since they are not expressed in the frontal appendages although their arthropod orthologs are expressed in the labrum (i.e., *rx*, *scro*, *FoxQ2*, *vsx/chx*) (Figs. 2, 3) [34, 37]. This does not only impede interpretation, but also cast doubts on the suitability of anteriorly expressed markers for the testing of the homology hypothesis concerning the labrum and the frontal appendages.

It remains unclear whether the observed differences in the onychophoran and/or arthropod lineage are a result of morphological change during the course of evolution, or whether the similar expression patterns in the labrum and the frontal appendages merely are the result of convergent evolution and the recruitment of a similar set of genes or a remnant of homology. We have to understand the function and the gene regulatory network in which *six3* is involved better to make convincing statements about its potential relevance as homology criterion.

## Conclusions

This study shows that the gene expression landscape in the anterior head of onychophorans and arthropods does not unambiguously support homology of the arthropod labrum with the frontal appendages (=primary antennae) of onychophorans. Instead, the new data reveal that only some of the genes that are expressed in the labrum are also expressed in the frontal appendages. Thus, it is not justified to take a single gene that is expressed in both structures as strong evidence for homology.

## Additional files

**Additional file 1: Table S1.** Primer sequences.

**Additional file 2: Table S2.** Accession numbers for genes used in the phylogenetic analysis.
