# Supplementary material for: Comparative analysis of gene expression patterns in the arthropod labrum and the onychophoran frontal appendages, and its implications for the arthropod head problem
Source: EvoDevo. 2017 Jan 3;8:1. doi: 10.1186/s13227-016-0064-4 (PMC5209905; doi:10.1186/s13227-016-0064-4)
Supplement: Supplementary file 1 — Additional file 1: Table S1. Primer sequences. [file 13227_2016_64_MOESM1_ESM.docx]

| gene | fw primer | bw primer | length |
| --- | --- | --- | --- |
| *Ek-hbn* | AAACGCAACTGAGTCC | CCATACTGGCGGAATG | 990 bps |
| *Ek-FoxQ2* | CAGGTATGCTTTACCC | TGCCATCTTTCAGCCG | 1089 bps |
| *Ek-nkx2.1/scro* | CCACAACTTACCGTCCGA | TACGATGCCGAACACATG | 844 bps |
| *Ek-rx* | TACGGCAGAAAAGCG | GTTCTTTCGCCTTCATT | 806 bps |
| *Ek-vsx/chx* | CAAAGTAATCCTTCTGC | TGGACATGGTTTTGTTG | 957 bps |
| *Gm-hbn* | TTTGGACACCGAGGACATGG | GATTCAAGCTGGTAATTGCC | 1016 bps |
| *Gm-FoxQ2* | GTACAACTACGCTTACG | GTCAGGATCAGGTGCC | 730 bps |
| *Gm-nkx2.1/scro* | TGCTCTTCACCCAGGC | ACACCCATTTTGCTGC | 810 bps |
